# An extreme-phenotype genome‐wide association study identifies candidate cannabinoid pathway genes in *Cannabis*

**DOI:** 10.1038/s41598-020-75271-7

**Published:** 2020-10-29

**Authors:** Matthew T. Welling, Lei Liu, Tobias Kretzschmar, Ramil Mauleon, Omid Ansari, Graham J. King

**Affiliations:** 1 Southern Cross Plant Science, Southern Cross University, Lismore, NSW 2480 Australia; 2 La Trobe Institute for Agriculture and Food, Department of Animal, Plant and Soil Sciences, School of Life Sciences, La Trobe University, Melbourne, VIC 3086 Australia; 3 Ecofibre Ltd, Brisbane, QLD 4014 Australia; 4 Ananda Hemp Ltd, Cynthiana, KY 41031 USA

**Keywords:** Genome-wide association studies, Plant genetics

## Abstract

*Cannabis* produces a class of isoprenylated resorcinyl polyketides known as cannabinoids, a subset of which are medically important and exclusive to this plant. The cannabinoid alkyl group is a critical structural feature that governs therapeutic activity. Genetic enhancement of the alkyl side-chain could lead to the development of novel chemical phenotypes (chemotypes) for pharmaceutical end-use. However, the genetic determinants underlying *in planta* variation of cannabinoid alkyl side-chain length remain uncharacterised. Using a diversity panel derived from the Ecofibre *Cannabis* germplasm collection, an extreme-phenotype genome-wide association study (XP-GWAS) was used to enrich for alkyl cannabinoid polymorphic regions. Resequencing of chemotypically extreme pools revealed a known cannabinoid synthesis pathway locus as well as a series of chemotype-associated genomic regions. One of these regions contained a candidate gene encoding a β-keto acyl carrier protein (ACP) reductase (BKR) putatively associated with polyketide fatty acid starter unit synthesis and alkyl side-chain length. Association analysis revealed twenty-two polymorphic variants spanning the length of this gene, including two nonsynonymous substitutions. The success of this first reported application of XP-GWAS for an obligate outcrossing and highly heterozygote plant genus suggests that this approach may have generic application for other plant species.

## Introduction

*Cannabis* is a multi-use predominantly dioecious and highly heterozygote plant genus^1^ within the angiosperm family *Cannabaceae*^2^. Medicinal use of *Cannabis* is thought to have spanned several millennia^3^, although incorporation of plant-derived drug products into modern western medicine has been hampered for several decades due to the narcotic status of this plant^4,5^.

Therapeutic activity of *Cannabis* is associated with a class of secondary metabolites commonly identified as cannabinoids^6,7^. Mature plants are prolific producers of these compounds, which are predominantly synthesised in capitate stalked trichomes and subsequently accumulate as exudates within their storage cavities^8^. The United States Food and Drug Administration (FDA) recently approved the first *Cannabis*-based medicine^9^, a purified plant extract containing the cannabinoid cannabidiol (CBD). This compound represents one of more than 100 naturally occurring plant cannabinoids^10–12^. In addition to CBD and the intoxicant delta-(9)-tetrahydrocannabinol (THC) other ‘minor’ cannabinoids produced by *Cannabis* show promise as therapeutic molecules^13,14^. However, these usually occur *in planta* at much lower concentrations^15^.

Cannabinoids are isoprenylated resorcinyl polyketides^16^, which are fused from intermediates derived from polyketide and isoprenoid biosynthetic pathways^17,18^. They are synthesised in plants with a carboxylated resorcinyl core, although this is readily decarboxylated non-enzymatically^19^. The dicyclic CBDA-type and tricyclic THCA-type cannabinoids are formed from the cyclisation of the isoprenoid residue by cannabidiolic acid synthase (CBDAS) and delta-(9)-tetrahydrocannabinolic acid synthase (THCAS)^20,21^. The activity of these synthases determines the structure of cannabinoid ligands, and so their ability to modulate the endocannabinoid system and associated physiological effects^22^. The alkyl side-chain is a further critical structural feature that influences potential therapeutic activity^23,24^, with side-chain length ranging *in planta* from between one to seven carbons^25–29^. However, cannabinoids with a five-carbon alkyl side-chain typically predominate in contemporary domesticated plants^30–32^.

Although there is potential for restructuring metabolic networks so that novel recombinant chemical phenotypes (chemotypes) can be developed for biopharmaceutical end-use^33^, the genetic and biosynthetic regulation of the alkyl group is largely unknown. Biosynthesis of cannabinoids within engineered yeast strains has demonstrated the promiscuity of cannabinoid pathway enzymes and their ability to produce alkyl cannabinoid homologs with varying chain lengths and configurations^34^. However, the biosynthetic origin of polyketide fatty acid (FA) starter units that determine alkyl homology is not fully understood^35,36^. Moreover, the genetic components underlying alkyl cannabinoid chemotypes have not been characterised^37,38^. This limits application of modern genetic improvement strategies, including marker-assisted selection (MAS) and genome engineering^39^.

The past decade has seen a rapid expansion in the use of high-throughput sequencing approaches to elucidate secondary metabolism in plants^40–42^. These have included using whole-genome sequencing such as used in *Ocimum sanctum* (holy basil)^43^ or RNA-Seq as used for *Brassica juncea* (Indian mustard)^44^. More targeted sequencing-based strategies in *Nicotiana tabacum* (tobacco)^45^ and *Curcuma longa* (turmeric)^46^, have contributed to improving the accuracy with which chemotype can be genetically characterised and predicted. However, the effectiveness of some of these methodologies can be limited in non-model orphan plant species^47^, where the inbred research lines required for linkage-based analysis such mapping-by-sequencing (MBS)^48^ may not be available^49^. This is especially problematic in *Cannabis* where the ability to acquire and exchange ex situ genetic resources for research continues to be constrained by regulatory requirements^50^.

Association mapping is an alternative high-throughput sequencing approach that circumvents the requirement for a dedicated crossing experiment. This approach relies on historical recombination for detection of genetic intervals associated with a trait^51,52^, and has been demonstrated through genome-wide association analysis (GWAS) to provide greater resolution than would be achievable from similar sized family mapping populations^53^. However, GWAS can be prohibitively expensive when applied to large sets of individuals, an issue amplified in heterozygote organisms due to the requirement for a high sequencing depth per sample to determine genotype accurately^54^. Extreme-phenotype GWAS (XP-GWAS) has been developed as a novel solution to this problem and involves bulked segregant analysis (BSA) of phenotypes within a diversity panel, with plants being selected at the extremes of the trait distribution^52^. XP-GWAS has resolved QTL for kernel row number in *Zea mays* (maize)^52^ and has also been successfully applied to inbred lines of *Beta vulgaris* (sugar beet)^51^ and its crop wild relatives^55^ to identify trait-associated variants. More recently, this analysis has identified variants associated with caffeine and trigonelline content in the self-fertilising species *Coffea arabica* (Arabian coffee)^56^. However, few studies have evaluated the utility of XP-GWAS in obligate outcrossing and highly heterozygote plants such as *Cannabis*.

Here we demonstrate the potential for XP-GWAS to provide high-resolution gene level mapping using a globally representative *Cannabis* diversity panel. This was achieved using whole-genome resequencing of pools comprised of chemotypically extreme individuals, where pools were divergent for cyclic (dicyclic vs tricyclic) and alkyl (C_3_ vs C_5_ chain-length) cannabinoid composition. Mapping of reads to two *Cannabis* reference genomes and comparison of allele frequencies between bulked DNA pools allowed detection of a known cannabinoid synthase locus and de novo discovery of polymorphic regions harbouring putative candidate genes linked to alkyl side-chain length.

## Results

### Alkyl-cannabinoid profiling and demarcation of chemotypic pools

**Figure 1:**
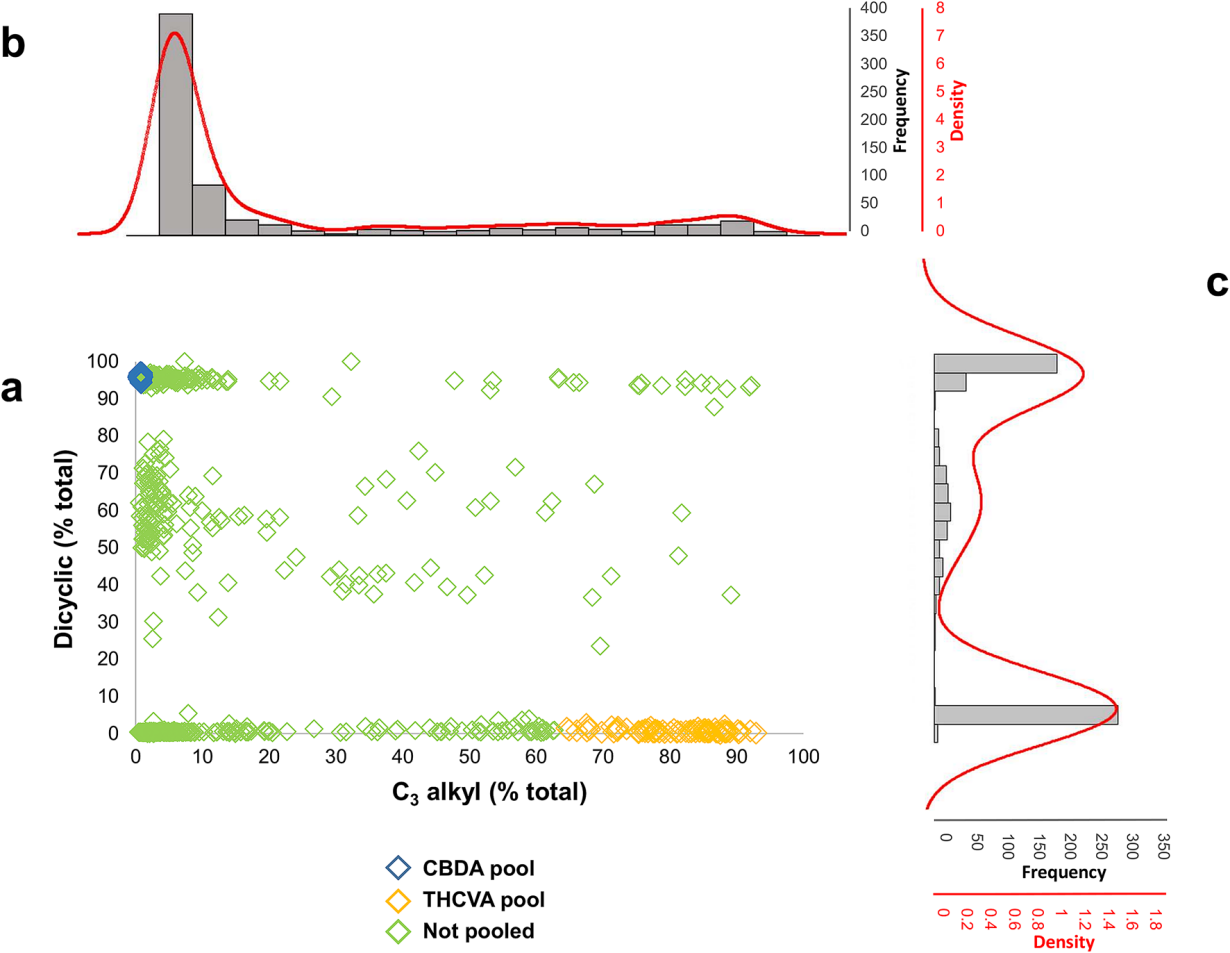
Chemotypic distribution of cyclic and alkyl cannabinoid composition in 711 *Cannabis sativa* L. plants. (**a**) Scatter plot showing distribution of bi-axial cannabinoid chemotypes. (**b**) Histogram and kernel density estimates of C_3_-alkyl cannabinoid chemotypes. (**c**) Histogram and kernel density estimates of dicyclic cannabinoid chemotypes. Plants within the diversity panel were selected from the Ecofibre Global Genetic Resource Collection. Cannabinoid composition was determined from liquid chromatography-mass spectrometry (LC–MS). Dicyclic and C_3_-alkyl cannabinoid fractions within the total cannabinoid fraction were calculated from fresh weight (*w/w*) cannabinoid content. Alkyl cannabinoid values are skewed towards low C3-alkyl, while dicyclic values exhibit a tripartite chemotypic distribution. *Blue diamond* (CBDA) and *yellow diamond* (THCVA) data points represent individual plants selected for an extreme-phenotype genome-wide association study.

We established a chemotype diversity panel from germplasm derived from the Ecofibre Global Germplasm Collection (EFGGC). Each accession in the panel is a provisional population, due to the heterozygous obligate outcrossing nature of *Cannabis*. Seven hundred and eleven juvenile *Cannabis sativa* L. plants derived from 72 accessions were chemotyped. The relative proportions of C_3_-/C_5_-alkyl cannabinoids and di-/tri-cyclic cannabinoids were calculated as a percentage of total cannabinoid content from fresh plant material, with fresh weight concentrations determined from liquid chromatography–mass spectrometry (LC–MS) analysis (Fig. 1). The alkyl cannabinoid values of the diversity panel were skewed towards the wild type pentyl cannabinoid chemotype, while cyclic cannabinoid values exhibited a tripartite chemotypic distribution comprised of low, intermediate and high dicyclic values (Fig. 1).

The frequency of individuals in the diversity panel that exhibited chemotypically extreme values was sufficient to enable the pooling of plants divergent for both alkyl and cyclic chemotypes (Fig. 1, Supplementary Table S1). Two pools were constructed, each with seventy individuals that either exhibited [low C_3_-alkyl + high dicyclic (e.g. CBDA)] or [high C_3_-alkyl + low dicyclic (e.g. THCVA)] values at the extreme ends of alkyl and cyclic cannabinoid chemotypic distributions (Fig. 1). Each pool had a selectivity of 9.8% (Supplementary Fig. S1). For the pentyl dicyclic CBDA pool, values ranged from 94.3–97.1%, with C_3_ alkyl values ranging from 0.5–0.9% (Fig. 1, Supplementary Fig. S1), while those of the propyl tricyclic THCVA pool ranged from 0.1–2.5% and 64.6–92.9%, respectively.

### DNA sequencing and mapping

Bulked DNA from each chemotypically extreme pool was subject to whole genome sequencing with Illumina sequencing-by-synthesis technology, generating 250,996,133 paired end (PE) reads for the CBDA pool and 238,918,478 PE reads for the THCVA pool (Table 1). For each pool, reads were mapped to *Cannabis* var. Finola (FN) and *Cannabis* var. Purple Kush (PK) genomes, representative of dicyclic CBDA and tricyclic THCA pentyl alkyl cannabinoid chemotypic lineages, respectively. For the FN reference sequence, mean sequencing depths were 53.8 × for the CBDA pool and 52.7 × for the THCVA pool (Table 1). Since the PK reference sequence had a smaller haploid genome length (0.9 Gb for PK vs 1.0 Gb for FN), depth of coverage for PK mapped reads was higher for the CBDA (66.1 ×) and THCVA pools (63.2 ×) (Table 1). Breadth of coverage based on a mean sequencing depth ≥ 15 ×, averaged 73.5% (± 2.1) for the FN reference and 78.9% (± 0.4) for the PK reference (Table 1).

**Table 1:** Sequencing and alignment statistics.

Sample (pool)	Reads^a^	*Cannabis* var. Finola reference			*Cannabis* var. Purple Kush reference		
		Reads aligned^b^	Depth of coverage^c^	Breadth of coverage (%)^d^	Reads aligned^b^	Depth of coverage^c^	Breadth of coverage (%)^d^
CBDA	501,992,266	497,228,282	53.76	75.00	494,924,047	66.11	79.21
THCVA	477,836,956	473,117,256	52.65	72.05	471,191,752	63.19	78.64

Haploid genome length for concatenated *Cannabis sativa* L. var. Finola reference [GenBank: ASM341772v2] is 1,010,967,789 bp; haploid genome length for the concatenated *Cannabis sativa* L. var. Purple Kush reference [GenBank: ASM23057v2] is 900,956,908 bp; reference scaffolds concatenated into separate supper scaffolds using ScaffoldStitcher (see “Methods”); CBDA: cannabidiolic acid; THCVA: tetrahydrocannabivarinic acid.

^a^Total number of reads including the first and second read of pairs; reads sequenced according to the 2 × 125 bp (550 bp insert) paired end (PE) scheme.

^b^Total number of reads including the first and second read of pairs passing Illumina’s filter that aligned to the reference sequence.

^c^Mean sequencing depth (mean number of mapped reads that cover each base of the reference sequence).

^d^Percentage of bases of the reference sequence covered with mapped reads at a sequencing depth ≥ 15 ×.

Variant calling resulted in 25,285,455 (FN) and 23,360,033 (PK) short variants (SNP and InDels) when bulked pools were mapped to the reference sequences. Following hard filtering and removal of multiallelic variations and variant sites lacking genotypes for both chemotypic pools, two sets of high confidence biallelic variants were obtained for FN and PK mapped reads (Table 2). Post-call filtering identified 9,133,504 SNPs and 2,593,786 InDels when bulked pools were mapped to the FN reference genome, while a total of 7,926,104 SNPs and 2,714,230 InDels were identified from post-call filtering of PK mapped reads (Table 2). Allele frequency estimates (AFe) at each variant site were then determined for CBDA and THCVA bulked pools. An AFe of 0 indicates 100% of reads support the reference allele, while an AFe of 1 indicates 100% of reads support the alternative allele. To allow variant comparison of pools, we calculated per site delta-AFe values as the absolute difference in AFe. A delta-AFe value ≥ 0.9 was used to indicate homozygote dissimilarities between pools. Low coverage contributes to higher error in determining allele frequencies due to stochastic effects while high coverage indicates the possibility of collapsed repetitive sequences. To reduce erroneous AFe values, only delta-AFe values supported by 0.75–2.5 × the average depth of coverage were included.

**Table 2:** Number of short variants called using extreme-phenotype bulk pools when mapped to Finola and Purple Kush *Cannabis* reference sequences.

Reference	Variant	Raw^c^	Filtered^d^
*Cannabis sativa* L. var. Finola^a^	SNP	19,500,079	9,133,504
	InDel	5,785,376	2,593,786
	TOTAL (SNP + InDel)	25,285,455	11,727,290
*Cannabis sativa* L. var. Purple Kush^b^	SNP	17,483,802	7,926,104
	InDel	5,876,231	2,714,230
	TOTAL (SNP + InDel)	23,360,033	10,640,334

*SNP* single nucleotide polymorphism, *InDel* small insertion-deletion.

^a^Concatenated *Cannabis sativa* L. var. Finola reference sequence [GenBank: ASM341772v2].

^b^Concatenated *Cannabis sativa* L. var. Purple Kush reference sequence [GenBank: ASM23057v2].

^c^Raw unfiltered variants called using GATK HaplotypeCaller.

^d^Hard filtered biallelic variants with depth of coverage (DP) ≥ 15 × and genotype quality (GQ) ≥ 20 that had genotype calls for both chemotypic pools at the variant site.

### Chemotypic pools are able to detect the known *CBDAS* locus

Bulked DNA from individual plants with divergent cannabinoid values allowed genome-wide variant comparison for alkyl and cyclic cannabinoid chemotypes (Fig. 1). Prior knowledge of the location of the *CBDAS* locus in the *CBDAS* homozygote and chemotypically CBDA predominant FN genome served as a control for our XP-GWAS facilitated variant discovery in *Cannabis*. Genetic intervals represent resolvable intervals between two marker loci. Those displaying delta-AFe values of 1 were delineated algorithmically by plotting contiguous allele frequencies of the CBDA pool that matched the wild-type reference sequence (AFe ≤ 0.1), with deviant allele frequencies between the dicyclic CBDA pool and the mutant tricyclic THCVA pool introduced through chemotypic selection of pooled individuals. As such, fluctuating delta-AFe values within intervals were considered as variants exclusive to the tricyclic THCVA pool, which is comprised of individuals having only minor dicyclic cannabinoid levels (e.g. CBDA/CBDVA).

**Figure 2:**
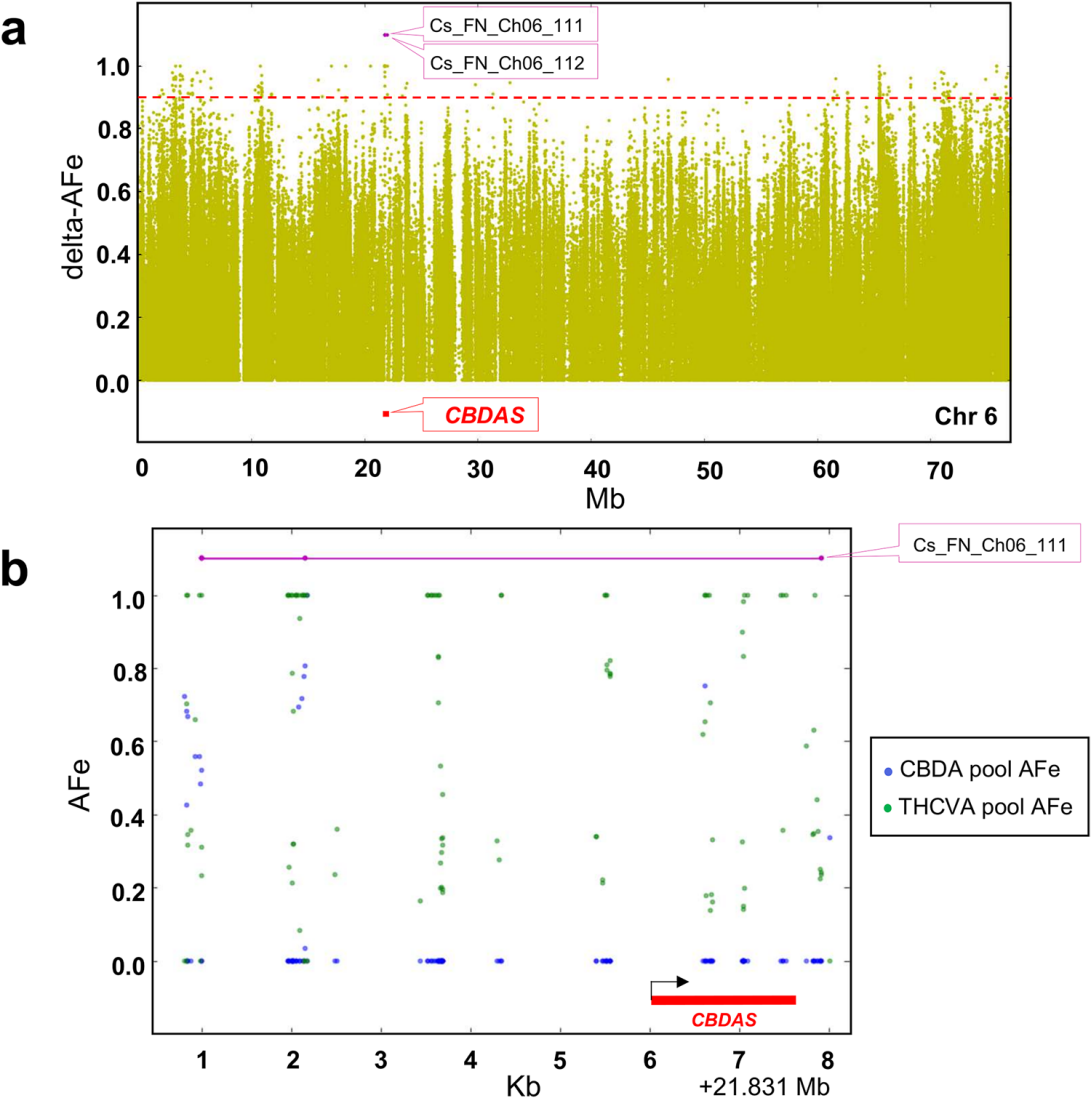
Detection of interval overlapping the *CBDAS* locus. (**a**) Plot of delta-AFe values encompassing two genomic regions (intervals) located within proximity to the chemotype determining *CBDAS* locus. AFe sorted by coordinate along chromosome six of the *Cannabis sativa* L. var. Finola (FN) genome. Delta-AFe indicates the absolute difference between AFe of CBDA and THCVA pools. *Red dotted line* indicates delta-AFe of 0.9. (**b**) Plot of AFe for the interval overlapping the 1632-nucleotide open reading frame of *CBDAS*. To reduce background noise, only delta-AFe supported by 0.75–2.5 × the average depth of coverage was plotted. *Black arrow* indicates orientation of gene (+/− strand). *Pink line* indicates genomic interval. Intervals displaying delta-AFe values of ≥ 0.9 were delineated using an algorithm which plots contiguous AFe values ≤ 0.1 in the CBDA pool (see “Methods”); AFe: allele frequency estimates; *CBDAS*: gene encoding cannabidiolic acid synthase.

**Figure 3:**
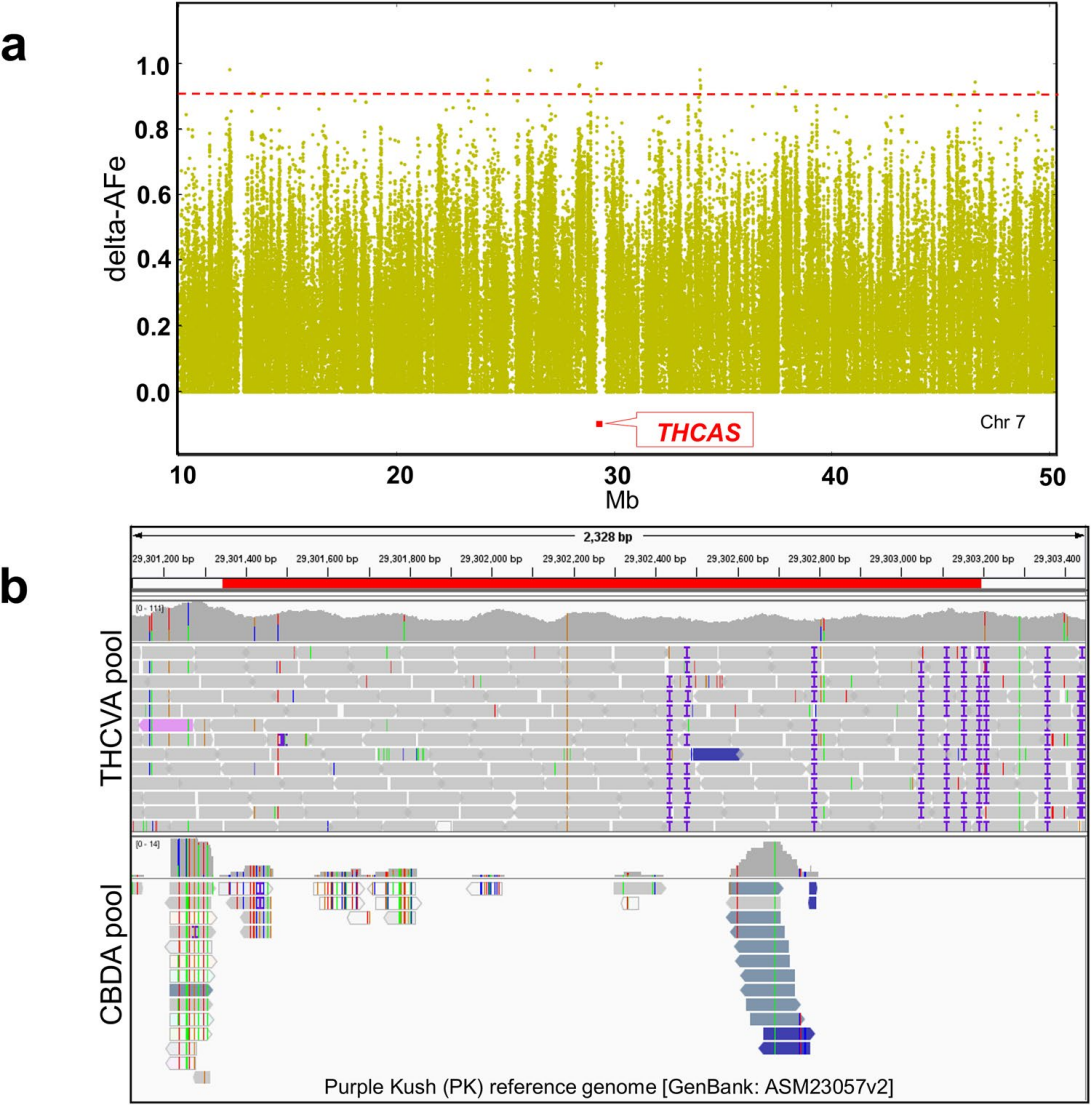
Non-homologous genomic region encompassing the *THCAS* locus. (**a**) Delta AFe values form a gap that overlaps the coordinates of the *THCAS* locus. delta-AFe is the absolute difference between AFe of CBDA and THCVA pools. (**b**) Integrative Genomics Viewer (IGV) snapshot of the *THCAS* locus. Reads from the THCVA pool were mapped to the PK reference sequence but reads for this region were absent from the CBDA pool; delta-AFe plotted by coordinate along chromosome seven of the *Cannabis sativa* L. var. Purple Kush (PK) genome. *Bold red line* indicates position of the *THCAS* locus. *Red dotted line* indicates delta-AFe of 0.9. *AFe* allele frequency estimates, *THCAS* gene encoding delta(9)-tetrahydrocannabinolic acid synthase.

Consistent with the known genomic position of the *CBDAS* locus, two intervals were detected on chromosome six sensu [GenBank: ASM341772v2] (Fig. 2a). One of these was coincident with the 6924 bp region that incorporates the 1632-nucleotide open reading frame (ORF) of *CBDAS* (Fig. 2b, Supplementary Table S2), with the *CBDAS* locus allele variant corresponding to GenBank accession AB292682.1 (100% query cover, 99.4% identity)*.* THCVA variants were supported by a mean depth of coverage of 156 × (compared with an average of 58 × for the CBDA pool) along the length of the *CBDAS* locus, with a subset of THCVA pool variants > 250 × coverage. Higher read coverage in the THCVA pool is consistent with multiple collapsed *CBDAS* gene copies being mapped to this locus. In total, 43 short variants (SNPs and InDels) with AFe values ≥ 0.9 were identified in the ORF of *CBDAS* (Supplementary Table S2). No intervals were detected which overlapped the coordinates of the *THCAS* locus when comparing chemotypic pools using the PK genome. However, a large gap (~ 350 kb) in delta-AFe values was detected in the middle of chromosome seven sensu [GenBank: ASM23057v2] (Fig. 3a). This region encompasses the coordinates of *THCAS* and this region was confirmed as non-homologous between pooled samples from raw alignment data (Fig. 3b). Reads from the THCVA pool mapped to the *THCAS* locus, while those from the CBDA pool failed to align to this region (Fig. 3b).

### Intervals detect putative candidate alkyl cannabinoid genes

**Figure 4:**
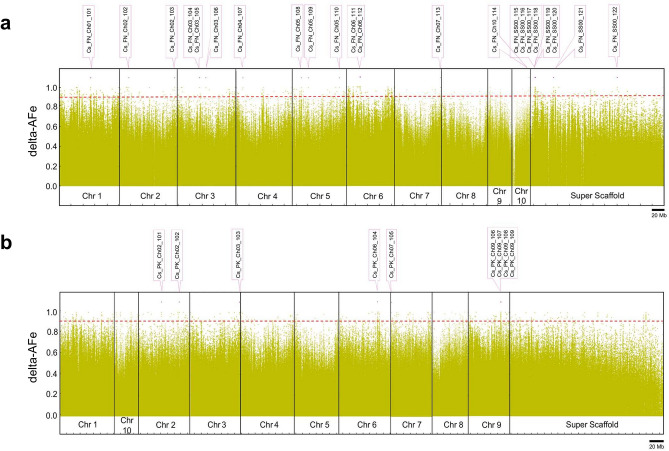
Delta-AFe between chemotypic pools and interval detection. (**a**) Delta-AFe plotted by chromosome number and coordinate along the *Cannabis sativa* L. var. Finola (FN) genome. Delta-AFe is the absolute difference between allele frequency estimates of CBDA and THCVA DNA pools. (**b**) Delta-AFe plotted by chromosome number and coordinate along the *Cannabis sativa* L. var. Purple Kush (PK) genome. Intervals displaying delta-AFe values of ≥ 0.9 were delineated using an algorithm which plots contiguous AFe values ≤ 0.1 in the CBDA pool (see “Methods”). To reduce background noise, only delta-AFe supported by 0.75–2.5 × the average depth of coverage was plotted. *Pink lines* indicate location of intervals. *Red dotted line* indicates delta-AFe of 0.9; *AFe* allele frequency estimates.

We next focused on interval detection for alkyl side-chain length. AFe values from the high pentyl alkyl CBDA pool and the high propyl alkyl THCVA pool were plotted for the remaining chromosomes and unplaced scaffolds for the genomes of FN and PK, both of which have a pentyl alkyl cannabinoid chemotype. To allow ad-hoc identification of genetic intervals relevant to alkyl cannabinoid chemotypes, AFe values were plotted from the CBDA pool which is chemotypically uniform for pentyl alkyl cannabinoids and therefore expected to match the reference sequence at sites determining alkyl chain length. Contiguous AFe values ≤ 0.1 from the CBDA pool revealed twenty-two intervals within the FN reference sequence (Fig. 4a), and nine intervals within the PK genome (Fig. 4b). Intervals ranged from 1–57 kb, with an average length of 10.3 kb (Supplementary Table S3, 4), and had a random distribution within both reference assemblies (Fig. 4a,b).

**Figure 5:**
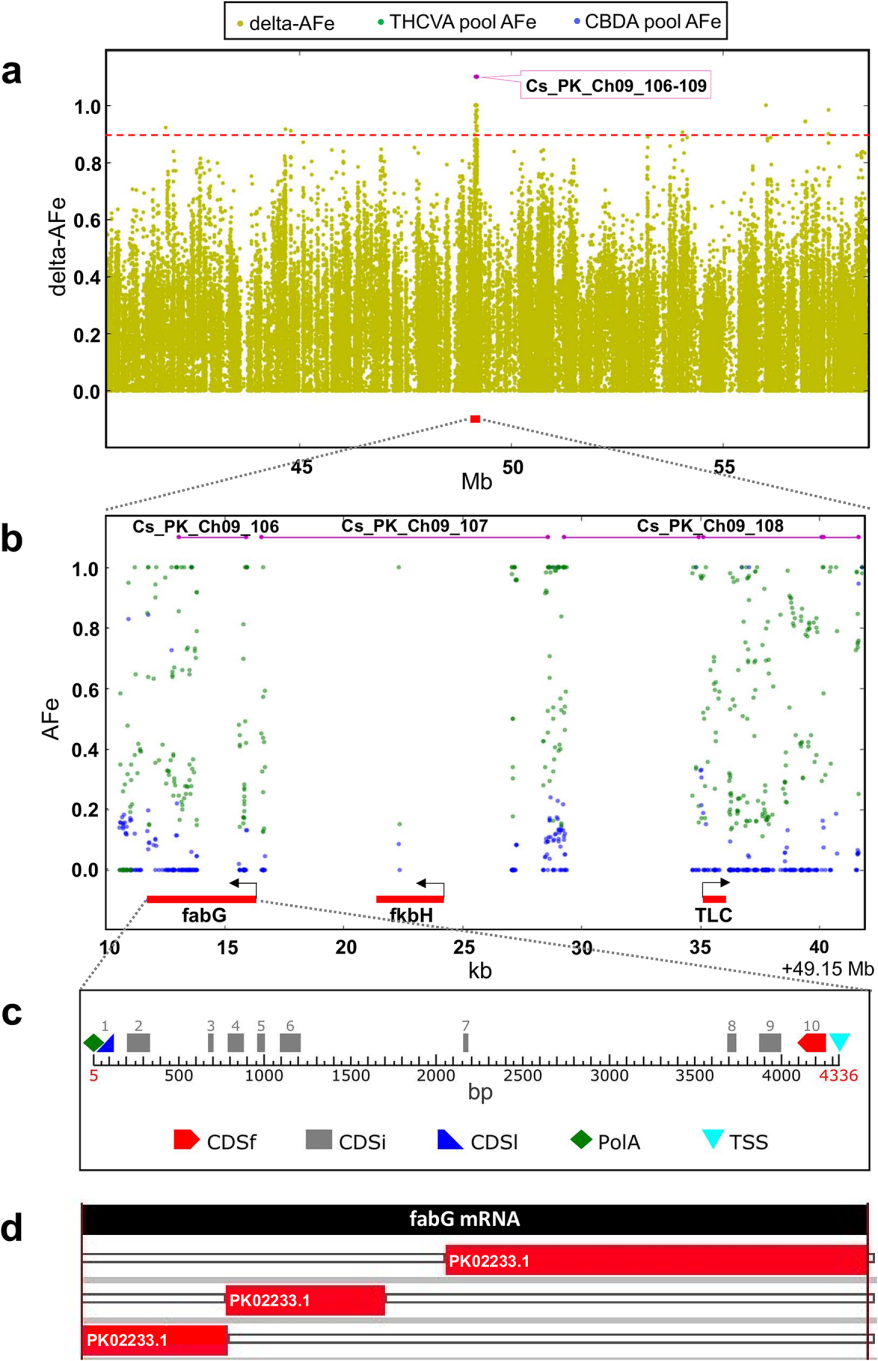
Genomic region encompassing putative candidate alkyl cannabinoid pathway genes. (**a**) Plot of delta-AFe within a region of chromosome nine on the *Cannabis sativa* L. var. Purple Kush (PK) genome. *Red dotted line* indicates delta-AFe of 0.9. *Pink lines* indicate location of intervals. *Red line* indicates location of putative candidate genes. (**b**) AFe of CBDA and THCVA pools under intervals. *Pink lines* indicate location of intervals. *Red line* indicates location of putative candidate genes. *Black arrow* indicates orientation of gene (+/− strand). (**c**) Positions of exons and gene features for the predicted *BKR* homolog gene. Gene features: CDSf, first exon including start codon; CDSi, internal exon; CDSl: last exon including stop codon; TSS, transcription start. Graphical output generated using FGENESH (v 2.6) (Softberry, http://www.softberry.com/berry.phtml)^87^. (**d**) Alignment of the mRNA sequence of the putative *BKR* gene homolog with the Purple Kush canSat3 draft representative transcriptome. High scoring alignments were found with segments of the PK transcript PK02233.1 (Supplementary Table S6). Graphic output generated using an in-house TimeLogic DeCypher system (Active Motif Inc., Carlsbad, CA) and TimeLogic Tera-BLASTN algorithm (v2.2.29) (Active Motif Inc., Carlsbad, CA) (https://www.timelogic.com).

At the time of analysis, a stable annotated reference sequence for *Cannabis* was unavailable. Genic regions within intervals were identified using FGENESH (v 2.6), with Basic Local Alignment Search Tool (BLAST)p alignments of predicted protein sequences used to determine potential gene function. Intervals were principally comprised of transposable element-like sequences (Supplementary Table S3, 4). However, PK intervals 106 (2.8 kb), 107 (12.1 kb) and 108 (12.4 kb) clustered on chromosome nine (Figs. 4b, 5a) and revealed three putative candidate genes (Fig. 5b). One of these encoded a protein with high identity to a β-keto acyl carrier protein (ACP) reductase (BKR; EC 1.1.1.100) (Supplementary Table S4). This protein has 81.7% identity to BKR homologs (80% query cover, *E*-value 4e−127) and incorporates a conserved fabG protein domain (NCBI accession PRK05557, *E*-value 1.44e−92). The two other putative candidate genes encoded a fkbH-like protein and a TLC domain-containing protein (NCBI accession smart00724, *E*-value 1.76e−13) (Fig. 5b, Supplementary Table S4). These intervals were not proximal to *CBDAS* and *THCAS* related sequences, and no copies of these genes were present on chromosome nine of the PK genome.

### Association analysis reveals putative alkyl cannabinoid-linked polymorphic variants

Analysis of polymorphisms within the three putative candidate genes revealed 25 variant sites with delta-AFe values ≥ 0.9, of which 22 spanned the length of the predicted BKR gene homolog located on PK interval 106 (Fig. 5b,c, Supplementary Table S5). Three of the *BKR* associated SNPs were in the 5′ untranslated region (UTR) and four were in exonic regions. Two of these were nonsynonymous and located within exon 6 (locus ID: *Cs*_BKR_06, *Cs*_BKR_07) (Supplementary Table S5, Fig. 5c). Nonsynonymous variants included a G/A SNP that would result in an Ala to Val substitution, as well as a T/C SNP that would result in a Ser to Asn substitution (Supplementary Table S5), with Val and Asn substitutions associated with the THCVA pool. Depth of coverage for the *BKR* homolog variant sites averaged 67 × for the CBDA pool and 74 × for the THCVA pool, with nonsynonymous SNPs supported by 62/61 reads for the CBDA pool and 65/64 reads for the THCVA pool (Supplementary Table S5). *BKR* nonsynonymous variants with delta-AFe values ≥ 0.9 did not overlap with the NAD(P) binding site or active site residues associated with the fabG conserved domain, and no other variant sites with AFe ≥ 0.9 were observed in exonic regions of the fkbH-like and TLC domain-encoding genes. However, mRNA sequences of putative candidate genes, including the *BKR* homolog (Fig. 5d), aligned to transcripts of the draft PK transcriptome (Supplementary Table S6).

## Discussion

To allow for the visual identification of genic regions under selection, an established algorithm which allows comparison between two phenotypically divergent DNA pools was used^51,55^. This has been successful in identifying the known *BvCYP76AD1* gene that encodes a cytochrome P450 enzyme responsible for betalain synthesis in *Beta vulgaris* (sugar beet)^51^. This algorithm has also been used to identify alleles of the *Rz2* gene which encodes a nucleotide-binding site leucine-rich repeat (NBS-LRR) protein conferring resistance against rhizomania disease in *Beta* crop wild relatives^55^. The criterion the algorithm uses for interval detection is a series of AFe values close to zero in the pool with the phenotype matching the reference sequence. This is calculated to allow a limited number of non-supportive reads (see “Methods”). Seed variants are used as starting locations for intervals, with intercepts (variants) not considered if they are flanked on each side by an AFe ≤ 0.1.

The algorithm for automatic interval detection was successful in identifying the location of the known *CBDAS* locus present on the FN genome (Fig. 2a,b). While the algorithm allows ad hoc identification of intervals, it does not assign a level of confidence. Given the lack of biological replicates for associations, calculating probability values for intervals is not straightforward. This could be addressed by sequencing individuals within pools or by sequencing a physical random pool to check for spurious associations^52^. However, both approaches would negate to some extent the lower costs associated with sequencing a maximum of two phenotypically extreme DNA pools^52,56^. One approach that could be assessed is to apply a Chi-squared test to the read counts for the pools surrounding intervals, in order to reject the null hypothesis that the phenotype and AFe are not correlated. As reported elsewhere^55^, a subset of intervals were fragmented over genic regions. This may be an indication that the tolerance for variants requires adjustment and that the algorithm is not exhaustive in its ability to capture genic sites associated with phenotype.

The genomic structure underlying dicyclic (CBDA/CBDVA) and tricyclic (THCA/THCVA) cannabinoid composition is now known to possess a high level of complexity and may vary considerably among different lineages and recombinants of *Cannabis*^37,38^. While segregation patterns support the hypothesis that cannabinoid synthases are isoforms at a single genetic locus *B*^57^, their genomic organisation appears to diverge significantly from this model^37,38,58^. Recent versions of the PK (tricyclic THCA-type) and FN (dicyclic CBDA-type) genomes, which were used for the current analysis, show two discrete loci that are non-homologous between chemotypes^37^. Within the PK assembly there is a *THCAS* locus, and in the FN assembly a *CBDAS* locus, neither of which has a clear counterpart in the opposite genome.

Analysis of AFe between CBDA and THCVA pools provides further insight into the genomic structure of the locus *B* complex. In our study, reads from the CBDA pool mapped to the *CBDAS* locus (FN genome), while no reads from the CBDA pool mapped to the *THCAS* locus (PK genome) (Fig. 3b). This is congruent with previous observations, where CBDA-predominant plants lack cannabinoid synthases gene copies that either align to the *THCAS* locus or that have > 95% nucleotide identity to functional *THCAS* sequences^37,58^. However, analysis of reads from the tricyclic THCVA pool showed differences in synthase heterogeneity as compared with the tricyclic PK genome. Previously, when sequence reads of the THCA predominant PK genome were mapped to the FN assembly, none aligned to the *CBDAS* locus^37^. In our analysis of the THCVA pool, not only did reads map to the *CBDAS* locus, but the THCVA pool yielded a high depth of coverage for this locus.

The tricyclic propyl alkyl THCVA chemotype is rarely found among domesticated forms of *Cannabis*, including those associated with medicinal^25^, industrial hemp^31,32,59^ and recreational drug end-uses^30^. One explanation for synthase heterogeneity between the PK genome and THCVA pool is that selection for THCA in PK has led to a loss in *CBDAS* gene copy number. High coverage of *CBDAS* by reads from the THCVA pool suggests that the current assemblies underestimate the total number of *CBDAS* gene copies that are present within the broader gene pool^37,38^. Low levels of the dicyclic cannabinoids (e.g. CBDA/CBDVA) in the tricyclic propyl alkyl THCVA pool would suggest that the *CBDAS* sequences from this pool are null alleles as evident by the presence of a 4 bp deletion previously associated with loss of function (Supplementary Table S2).

It is predicted that the sequencing of alternative bi-axial chemotypic pools THCA vs CBDVA would have provided additional insight into cannabinoid synthase heterogeneity and that this would have further validated the XP-GWAS approach through the identification of the *CBDAS* locus using independent bulked pools. However, this analysis was not possible due to the low representation of CBDVA chemotypes in the sample population. While not directly relevant to the chemotype selection of bulked pools, no intervals were detected which corresponded to *THCAS*-like *CBCAS* loci. These genes have 96% identical to *THCAS* at the nucleotide level^37^, although the position of these loci on unmapped scaffolds may have influenced mapping quality and subsequent variant analysis.

Although the reference genome sequences used are comprised of ten chromosome pseudomolecules, they remain fragmented with 2352 (FN) and 6295 (PK) unlocalised scaffolds^37^ and are predicted to contain unresolved THCA/CBDA loci^38^. While these and other near-complete haploid reference sequences are a substantial improvement on the previous draft genome^58^, development of a fully phased diploid assembly for *Cannabis* will accelerate understanding of haplotype structure and improve allele-specific analyses of complex loci such as those governing cannabinoid synthase activity. Given that evidence exists for linage-specific changes in cannabinoid synthase gene content^38^, development of ‘pan genomes’ should also aid in resolving the genetic molecular basis for cannabinoid composition among diverse subtaxa while also improving the analysis of pooled samples^60^.

^13^C labelling patterns in *Cannabis* have indicated that the alkyl group of cannabinoids originate from a fatty acid (FA) precursor^61^. This is also supported by experiments with engineered yeast strains fed various FA precursors, which result in altered production of cannabinoid alkyl homologs^34^. Polyketide FA starter units could arise via several metabolic routes. One hypothesis involves the breakdown of FAs by desaturases, lipoxygenases (LOX) and hydroperoxide lyases^36,58^. Alternative paths involve the breakdown of branched-chain amino acids, as occurs in prenylated polyketide synthesis in the closely related *Cannabaceae* species *Humulus lupulus* (hops)^62^, as well as the de novo FA synthesis pathway in *Cannabis*^35^. The latter two examples both involve BKR activity for the development of polyketide starter units^35,63^.

In type II FA synthesis, which occurs in plants and many bacteria, FAs are constructed from the condensation of two-carbon units by a series of discrete mono-functional enzymes: β-ketoacyl-ACP synthase (KAS), BKR, β-hydroxyacyl-ACP dehydrase (HAAD) and enoyl-ACP reductase (ENR)^64^. BKR catalyses the first reductive step in FA elongation using malonyl-CoA-derived building blocks^65^. Analogous with its function in de novo FA synthesis, this enzyme performs a similar reductive step in polyketide synthesis^66^. It shares significant sequence similarity with the second FA synthesis reductase ENR, as well as with other members of the short-chain dehydrogenases/reductases (SDR) family^66–68^. This is also found with the protein sequence of the predicted *BKR* homolog, with BLASTp analysis yielding a specific hit to the SDR family domain adh_short (GenBank accession pfam00106, *E*-value 6.56e−70).

The *BKR* homolog identified on PK interval 106 may have a putative role in the synthesis of polyketide FA starter units. However, it is difficult to speculate on the mechanism by which this gene contributes to alkyl side-chain length without biochemical examination of its encoded protein. This is due to the similarity of BKR with other oxidoreductases of the SDR superfamily^69^, as well as limited structural data on the transient complexes BKR forms with other FA synthesis proteins^70^. As with KAS^71^ and ENR^72^ type II FA synthesis enzymes, BKR (fabG) isoforms have demonstrated the ability to shift FA composition^67^. In *Capsicum*, the *CaKR1* gene has been found to encode a BKR which facilitates FA elongation of the capsaicinoid intermediate 8-methyl-6-nonenoic acid^63^, with *CaKR1* gene silencing influencing capsaicinoid anabolism. The role of *BKR* in cannabinoid biosynthesis is also supported by quantitative PCR (qPCR) analysis, where *BKR*-associated unigenes were found to be expressed at high levels in trichome *vs* leaf tissue^35^. In addition, the *BKR* homolog identified on PK interval 106 aligned to the draft PK transcriptome, with 91% query cover and ≥ 99% identity with segments of the PK02233.1 transcript (Supplementary Table S6, Fig. 5d).

Other predicted candidate genes found clustered within 25 kb of the *BKR* homolog also have interesting putative functions relating to polyketide and FA synthesis. Proteins containing the fkbH domain are associated with the formation of glyceryl-ACP, where they function as part of a larger protein cluster involved in the synthesis of methoxymalonyl-ACP polyketide extender units^73,74^. TLC (TRAM/LAG1/CLN8) lipid-sensing domain-containing proteins are associated with several functions including acyl-CoA-dependent ceramide synthesis^75^ and lipid transfer^76^. PK and other high-yielding cannabinoid strains can produce small quantities of cannabinoids with varying alkyl-chain lengths including C_3_-alkyl homologs^26,29^. Contemporary industrial hemp cultivars such as FN, which are thought to have been derived from a narrow subset of the genepool and are more uniform in chemotype^30,50^, are principally associated with CBDA production^21,59^. This may explain why analysis of pooled chemotypes against the FN genome were not meaningful in the detection of the putative alkyl cannabinoid gene clusters.

While it is not obvious how intervals harbouring transposable element-like sequences are relevant to alkyl cannabinoid composition (Fig. 4a,b, Supplementary Table S3, 4), they may have utility in determining phylogeny and genetic diversity between chemotypic lineages or act as markers to tag and track traits of interest^77,78^. This information could be used to inform ex situ germplasm conservation and to develop representative core collections^79^.

We successfully demonstrated the application of a modified version of the GWAS approach with sequencing of bulked pools derived from a diversity panel of *Cannabis*^51,52^, an obligate outcrossing and highly heterozygote plant genus^1,80^. The approach was validated through the identification of a known complex locus involved in the synthesis of the dicyclic cannabinoid CBDA^37^, and has led to the de novo identification of putative candidate cannabinoid pathway genes. Resequencing of pools divergent for both CBDA and THCVA allowed genetic characterisation of two distinct chemotypes, resulting in the detection of trait-associated variants within a candidate gene putative linked to alkyl cannabinoid synthesis. This study made use of rare allelic variation present within a subset of the *Cannabis* genepool and highlights the importance of ex situ germplasm conservation and the systematic phenotyping of these resources for genetic improvement^50,81^.

The analysis presented here offers insight into the biosynthesis of alkyl cannabinoids and provides a platform for further genetic characterisation of alkyl cannabinoid metabolism. The putative variants located within intervals and their flanking sequences provide useful templates for the development of PCR-based markers or as probes for targeted high-throughput DNA sequencing. This approach could be applied to analysing progeny of a segregating population or used to screen germplasm with intent to develop marker haplotypes for alkyl-cannabinoid composition. The outcome of this analysis also provides putative targets for gene editing and other interventions, including opportunities to validate trait-associated genetic regions and associated candidate genes using reverse genetic approaches.

## Methods

### Genetic materials and cultivation

All research activities, including the procurement and cultivation of the prohibited plant *Cannabis*, were executed in accordance with the NSW Drug Misuse and Trafficking Act 1985 and under authorisations granted to Professor Graham King by the New South Wales Ministry of Health, Pharmaceutical Regulatory Unit, Legal and Regulatory Services Branch, Australia. *Cannabis sativa* L. seed pack accessions were sourced from the Ecofibre Global Germplasm Collection. Seventy-two accessions were used to develop a chemotypic diversity panel comprising of 711 individual plants. Dr. Omid Ansari (omid@ecofibre.com) is the contact person for enquiries on germplasm used in this study and the associated Ecofibre ex situ *Cannabis* genetic resource collection.

Seeds were sown directly into 7.5 cm (diameter) × 10 cm (height) 400 mL round pots at a depth of 1.5 cm. Soil media consisted of one part perlite, one part peat moss, and one part vermiculite as well as dolomite at a concentration of 110 g/100 L. Plants were grown under environmentally uniform conditions within Ecofibre’s purpose-built growth chambers, and were subject to 11 h of high pressure sodium (HPS)/metal halide (MH) light (luminous flux = 72,000 lumens) per day. Temperature was kept constant between 26 and 28 °C. Plants were watered daily. Upon full extension of the first leaflet pair plants were supplemented with CANNA Aqua Vega solution.

### LC–MS chemotyping

Cannabinoids were extracted from the sub-apical raceme of juvenile plants (code 1008)^82^ at opposing phyllotaxis (2 × 250 mg) and analysed using liquid chromatography-mass spectrometry (LC–MS) as previously described^31^. Dicyclic, tricyclic, C_3_-alkyl and C_5_-alkyl cannabinoid fractions within the total cannabinoid fraction were calculated from the fresh weight (*w/w*) content of cannabidivarinic acid (CBDVA), tetrahydrocannabivarinic acid (THCVA), cannabidiolic acid (CBDA), delta(9)-tetrahydrocannabinolic acid (THCA), cannabidivarin (CBDV), delta(9)-tetrahydrocannabivarin (THCV), cannabidiol (CBD) and delta(9)-tetrahydrocannabinol (THC).

### DNA isolation, library preparation and sequencing

DNA was isolated from each plant prior to bulking. DNA extraction and purification involved using a Qiagen DNeasy Plant Mini Kit in accordance with the manufacturer’s instructions, with tissue disruption performed using a Qiagen TissueLyser. A Qubit Fluorometer and Qubit dsDNA BR Assay Kit was used for DNA quantification. DNA concentration (*w/v*) in Qiagen AE buffer (10 mM Tris–Cl 0.5 mM EDTA; pH 9.0) was normalised, pooled and concentrated to 129 μg/μL using an Eppendorf Concentrator #5301 with diaphragm vacuum pump. Molecular weight (≥ 20 kb) and RNA contamination were assessed by gel electrophoresis.

Library preparation and sequencing of pooled DNA samples was undertaken at the Australian Genome Research Facility Ltd (AGRF, Melbourne, Australia; https://agrf.org.au). DNA libraries were prepared using an Illumina TruSeq DNA PCR-Free Library Prep Kit, with DNA fragmentation, end repair, size selection, A-tailing and adapter ligation conducted according to the manufacturer's guidelines. Whole-genome sequencing of DNA pools was performed on an Illumina HiSeq2500 platform in high-output mode. Samples were run over two flow cells and sequenced according to the 2 × 125 bp (550 bp insert) paired end (PE) scheme using HiSeq HT chemistry. Image analysis was performed by HiSeq Control Software (HCS, v2.2.68) and Real Time Analysis (RTA, v1.18.66.3). Data was generated using the bcl2fastq 2.20.0.422 pipeline.

### Data pre-processing and mapping

All data pre-processing and short variant discovery steps were performed using the Genome Analysis Toolkit (GATK, v4.0.3.0)^83^. Sequence reads of pooled samples were separately mapped to modified versions of *Cannabis sativa* L. var. Finola (FN) [GenBank: ASM341772v2] and var. Purple Kush (PK) [GenBank: ASM23057v2] reference assemblies^37^. Modified reference sequences were developed using the tool ScaffoldStitcher (Python) (https://bitbucket.org/dholab/scaffoldstitcher/src). ScaffoldStitcher concatenated unplaced scaffolds using spacers of 550 Ns.

Analysis-ready Binary Alignment Map (BAM) files were developed with consideration of the Broads GATK Best Practices workflows (https://gatk.broadinstitute.org). FastqToSam was used to generate unmapped BAM (uBAM) files. Adapter sequences were tagged using MarkIlluminaAdapters. uBAMs were reverted to FASTQ and purged of metadata using SamToFastq. Reads were then mapped to modified FN and PK reference assemblies using Burrows-Wheeler Aligner's maximal exact matches (BWA-MEM, v0.7.17) algorithm^84^. Duplicates were tagged using MarkDuplicates. Pooled sample BAM files were sorted by coordinate with SortSam and validity using ValidateSamFile. Sequencing and alignment statistics were determined from CollectAlignmentSummaryMetrics. Depth and breadth of coverage were assessed using AWK, mpileup and depth commands (Sequence Alignment/Map (SAM)tools, v 1.7)^85^.

### Short variant discovery

Base Quality Score Recalibration (BQSR) was performed as outlined by GATK guidelines. Recalibration tables were written with BaseRecalibrator and ApplyBQSR was used to recalibrate BAM files. BQSR was bootstrapped and convergence of successive recalibration outputs assessed using AnalyzeCovariates.

Single nucleotide polymorphism (SNPs) and small insertion-deletion (InDels) were identified using the Broads GATK HaplotypeCaller. HaplotypeCaller runs were performed in GVCF mode, with chemotypic pool gVCF files consolidated using CombineGVCFs. Variants were joint called using GenotypeGVCFs. Variants were then hard filtered with SelectVariants and VariantFiltration tools in accordance with GATK generic filtering parameters. Variants were restricted to biallelic allelicity. Variants supported by a depth of coverage (DP) < 15 or a genotype quality (GQ) value < 20 were not considered. Sites with no-call genotypes that were deficient in one of two chemotypic pool genotypes were excluded. Bi-sample SNP and InDel VCFs were then merged using MergeVcfs to accommodate downstream analysis.

### Allele frequency estimation

Allele frequency estimates (AFe) were calculated at each site per DNA pool using the relative number of reads supportive of either the alternative or reference allele. An AFe of 0 indicates 100% of reads support the reference allele, while an AFe of 1 indicates 100% of reads support the alternative allele. To demarcate AFe values associated with either chemotypic pool, per site delta-AFe values were calculated as the absolute difference of AFe values between pools. Delta-AFe values ≥ 0.9 indicated homozygote differences between pools. AFe and delta-AFe values were calculated for biallelic variants and plotted by coordinate along FN and PK genome assemblies. To reduce false positive variants, only delta-AFe values supported by 0.75–2.5 x the average depth of coverage was plotted^51^.

### Interval detection and visualization

Genomic regions exhibiting AFe value ≥ 0.9 were delineated using an established algorithm for automatic interval detection^51,55^. Intervals were demarcated by a series of AFe values ≤ 0.1 in the CBDA pool. Interruptions by an intercept (variant) were ignored if they were comprised of a maximum of 1 variant with an AFe ≥ 0.1, flanked on both sides by a variant with an AFe of ≤ 0.1. The starting point of intervals was determined from seed variants exhibiting AFe minima values (≤ 0.1). AFe minima was calculated to permit a small number of non-supportive reads using *X* = (*P *– 100 × *E*/*C*), with bulked pool phenotypic difference *P* = 0.9, combined coverage of the pools *C* and an estimate sequencing error from the Illumina HiSeq platform *E* = 0.01. Variants overlapping intervals were then filtered for AFe ≥ 0.9. The Python script VCF2AFAnalysis.py (https://github.com/davidries84/vcf2AFAnalysis) was used to generate intervals and to plot AFe and delta-AFe values^51^. Visualisation of raw alignment data and regions of interest was performed using Integrative Genomics Viewer (IGV, v2.6.0) software^86^.

### Gene predication and annotation

Multifasta files containing interval sequences were indexed using faidx (SAMtools, v 1.7). Putative genes and associated protein sequences were predicted using FGENESH (v2.6) (https://www.softberry.com/)^87^. FGENESH was performed using *Cannabis*-specific gene finding parameters (date accessed 04/12/2019). For functional annotation of predicted genes, Basic Local Alignment Search Tool (BLAST)p analysis of amino acid sequences were performed against the non-redundant protein sequence database (National Center for Biotechnology Information (NCBI) nr protein, accessed 12/04/2019 containing 198,058,131 sequences)^88^. The highest scoring characterised protein was extracted, with consideration for identity, coverage, then *E*-value. Nucleotide sequences [mRNA] of putative candidate genes identified on PK intervals on chromosome nine were queried using an in-house TimeLogic DeCypher system (Active Motif Inc., Carlsbad, CA) and TimeLogic Tera-BLASTN algorithm (v2.2.29) (Active Motif Inc., Carlsbad, CA) (https://www.timelogic.com) against the canSat3 representative transcriptome (Cannabis Genome Browser, https://genome.ccbr.utoronto.ca/downloads.html), with an *E*-value cut-off limit of 1e−10 applied.

## Supplementary information

Supplementary Information 1.

Supplementary Information 2.

## Data Availability

The Illumina sequencing data for chemotypic extreme pools from this study have been submitted to the NCBI BioProject database under accession number PRJNA669610 (https://www.ncbi.nlm.nih.gov/bioproject). For enquiries relating to the Ecofibre germplasm collection and commercial arrangements contact Omid Ansari: omid@ecofibre.com.
