# Supplementary material for: An extreme-phenotype genome‐wide association study identifies candidate cannabinoid pathway genes in Cannabis
Source: Sci Rep. 2020 Oct 29;10:18643. doi: 10.1038/s41598-020-75271-7 (PMC7596533; doi:10.1038/s41598-020-75271-7)
Supplement: Supplementary file 2 — Supplementary Information 2. [file 41598_2020_75271_MOESM2_ESM.pdf]

Supplementary Information

An Extreme-Phenotype Genome-Wide Association Study Identifies Candidate Cannabinoid Pathway Genes in *Cannabis*

Matthew T. Welling[1,2], Lei Liu[1], Tobias Kretzschmar[1], Ramil Mauleon[1], Omid Ansari[3,4], Graham J. King[1,*]

[1]Southern Cross Plant Science, Southern Cross University, Lismore, New South Wales 2480, Australia.

[2]La Trobe Institute for Agriculture and Food, School of Life Sciences, Department of Animal, Plant, and Soil Sciences, AgriBio, La Trobe University, Bundoora, VIC, 3086, Australia.

[3]Ecofibre Ltd, Brisbane, Queensland 4014, Australia.

[4]Ananda Hemp Ltd, Cynthiana, Kentucky 41031, USA.

* Correspondence:

Graham J. King

graham.king@scu.edu.au

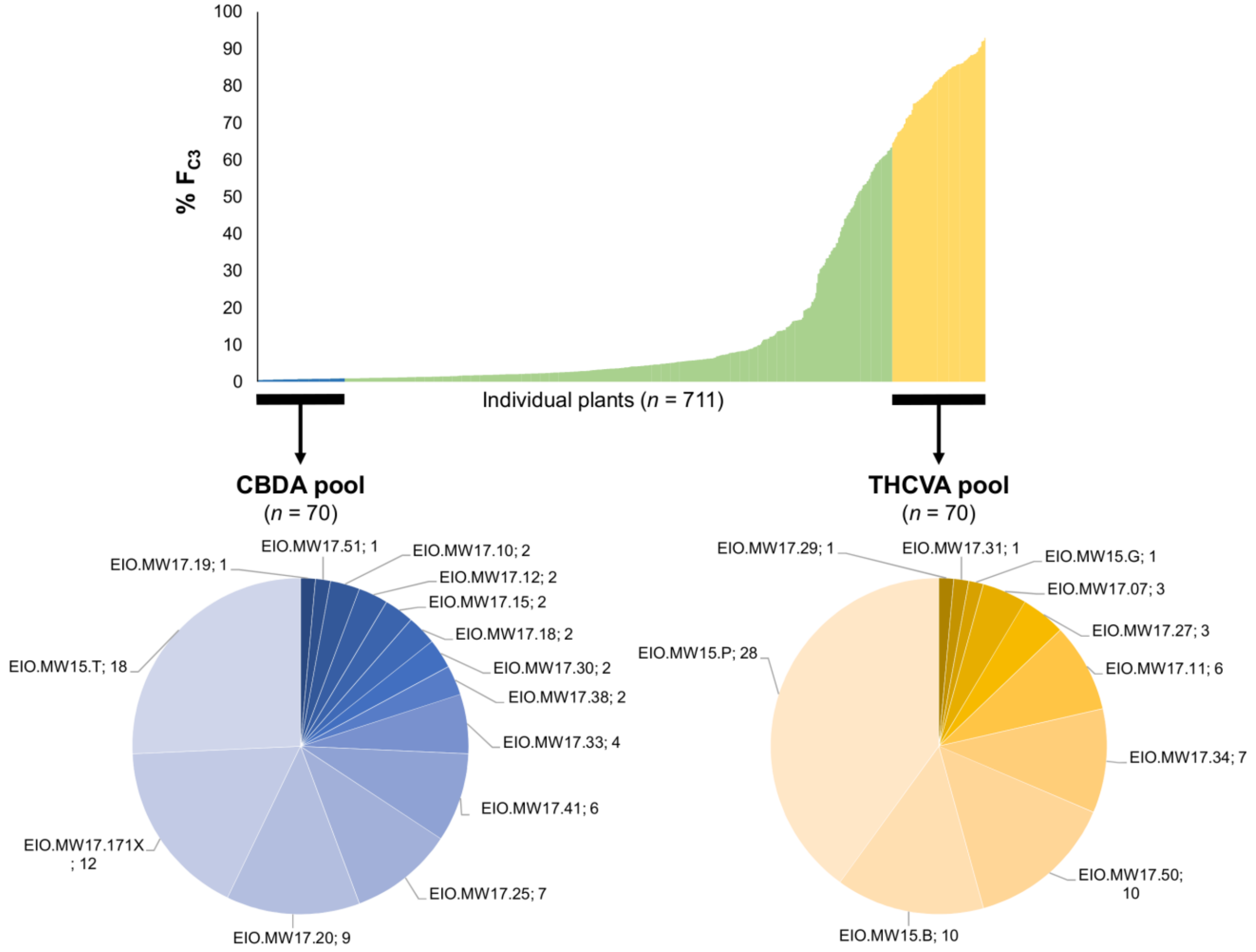

Supplementary Figure S1. Selection intensity of bulked pools used for an extreme-phenotype genome-wide association study. Alkyl cannabinoid values in 711 plants determined from liquid chromatography-mass spectrometry (LC-MS). C_3-alkyl cannabinoid fractions within the total cannabinoid fraction were calculated from fresh weight (*w/w*) cannabinoid content. Seventy individual plants per pool were used for an extreme-phenotype genome-wide association study, with a selectivity of 9.84 %. Individuals used in CBDA and THCVA chemotypically extreme pools were derived from 14 and 10 seed pack accessions, respectively. *Black bars* represent the range of C_3-alkyl cannabinoid values exhibited by plants used for bulk segregant analysis. *Pie charts* represent percentage of bulked plants per accession. *Number* after semicolon is the number of plants bulked per accession.

Supplementary Tables

Supplementary Table S2. Exon variant sites for *CBDAS*.

Chromosome	Position	Reference allele	Alternative allele	CBDA pool AFe	THCVA pool AFe	Delta-AFe
CM011610.1	21837002	C	T	0.0	1.0	1.0
CM011610.1	21837018	T	A	0.0	1.0	1.0
CM011610.1	21837092	T	C	0.0	1.0	1.0
CM011610.1	21837115	C	A	0.0	1.0	1.0
CM011610.1	21837168	A	C	0.0	1.0	1.0
CM011610.1	21837169	T	C	0.0	1.0	1.0
CM011610.1	21837174	C	T	0.0	1.0	1.0
CM011610.1	21837189	TCGTA	T	0.0	1.0	1.0
CM011610.1	21837210	C	A	0.0	1.0	1.0
CM011610.1	21837355	G	A	0.0	1.0	1.0
CM011610.1	21837370	T	C	0.0	1.0	1.0
CM011610.1	21837388	G	A	0.0	0.9	0.9
CM011610.1	21837444	G	A	0.0	1.0	1.0
CM011610.1	21837582	G	C	0.0	1.0	1.0
CM011610.1	21837625	C	T	0.0	1.0	1.0
CM011610.1	21837650	A	G	0.0	1.0	1.0
CM011610.1	21837674	C	G	0.0	1.0	1.0
CM011610.1	21837725	T	A	0.0	1.0	1.0
CM011610.1	21837741	C	G	0.0	1.0	1.0
CM011610.1	21837791	C	CCAA	0.0	1.0	1.0
CM011610.1	21837925	C	T	0.0	1.0	1.0
CM011610.1	21837956	G	A	0.0	1.0	1.0
CM011610.1	21838039	G	T	0.0	0.9	0.9
CM011610.1	21838054	G	A	0.0	1.0	1.0
CM011610.1	21838062	G	A	0.0	1.0	1.0
CM011610.1	21838099	T	C	0.0	1.0	1.0
CM011610.1	21838116	A	G	0.0	1.0	1.0
CM011610.1	21838132	C	T	0.0	1.0	1.0
CM011610.1	21838156	C	A	0.0	1.0	1.0
CM011610.1	21838221	T	C	0.0	0.9	0.9
CM011610.1	21838229	C	A	0.0	1.0	1.0
CM011610.1	21838260	T	A	0.0	1.0	1.0
CM011610.1	21838352	T	A	0.0	1.0	1.0
CM011610.1	21838360	G	A	0.0	1.0	1.0
CM011610.1	21838390	A	C	0.0	1.0	1.0
CM011610.1	21838399	C	A	0.0	0.9	0.9
CM011610.1	21838421	A	C	0.0	1.0	1.0
CM011610.1	21838427	A	G	0.0	1.0	1.0
CM011610.1	21838472	G	A	0.0	1.0	1.0
CM011610.1	21838490	G	C	0.0	1.0	1.0
CM011610.1	21838521	A	G	0.0	1.0	1.0
CM011610.1	21838524	C	A	0.0	1.0	1.0
CM011610.1	21838544	C	T	0.0	1.0	1.0

AFe: is the relative number of reads supportive of either the alternative allele or the reference allele. AFe of 0.0 indicates 100 % of reads support the reference allele. AFe 0f 1.0 indicates 100 % of reads support the alternative allele. AFe: allele frequency estimates; *CBDAS*: gene encoding cannabidiolic acid synthase; delta-AFe: absolute difference between AFe of CBDA and THCVA bulk pools.

Supplementary Table S3. Candidate genes identified from CBDA and THCVA pools using the FN reference sequence.

Interval (locus)	Length (bp)	Chr.	Start coordinate	Stop coordinate	Gene length (bp)	Predicted protein[1]	GenBank accession	Cover (%)	*E*-value	Identity (%)	Relevance t[2]
Cs_FN_Ch01_101	1,714	CM011605.1	53,067,046	53,068,760	552	Photosystem I PsaK, reaction centre	PON71667.1	34	7.9	30.30	*
Cs_FN_Ch02_102	6,276	CM011606.1	15,615,434	15,621,710	264	Uncharacterized protein LOC107061829	XP_015167212.1	50	5.9	36.36	*
					123	Dicer-like protein 4	XP_024928613.1	87	2e-14	94.29	*
Cs_FN_Ch02_103	1,007	CM011606.1	90,575,894	90,576,901	228	Retrovirus-related pol polyprotein from transposon RE1	RVW20207.1	60	1e-17	77.78	*
Cs_FN_Ch03_104	4,409	CM011607.1	36,220,499	36,224,908	2118	Retrovirus-related pol polyprotein from transposon TNT 1-94	RVX12712.1	93	0.0	50.65	*
Cs_FN_Ch03_105	22,595	CM011607.1	36,247,017	36,269,612	5304	Protein NYNRIN-like	XP_013650953.1	61	0.0	42.80	*
					891	B3 domain-containing protein Os01g0234100-like	XP_021751876.1	40	0.47	28.35	*
					948	Peptide methionine sulfoxide reductase	PON80230.1	43	9e-09	31.69	*
					195	Thylakoid ADP, ATP carrier protein, chloroplastic-like	XP_020218712.1	62	1.2	42.50	*
					363	Uncharacterized protein LOC111986372	XP_023873765.1	81	2e-10	38.32	*
Cs_FN_Ch03_106	12,343	CM011607.1	46,169,567	46,181,910	150	Hypothetical protein TorRG33x02_210200	PON83186.1	42	0.44	71.43	*
					303	Zinc finger, FYVE-type	OMO93156.1	77	3.1	30.00	*
					810	Transposon Ty3-I Gag-Pol polyprotein	RVW58909.1	96	5e-137	71.54	*
Cs_FN_Ch04_107	1,143	CM011608.1	11,031,676	11,032,819	N/A	No gene predicted for interval	N/A	N/A	N/A	N/A	N/A
Cs_FN_Ch05_108	1,547	CM011609.1	14,946,580	14,948,127	N/A	No gene predicted for interval	N/A	N/A	N/A	N/A	N/A
Cs_FN_Ch05_109	1,547	CM011609.1	14,946,580	14,948,127	N/A	No gene predicted for interval	N/A	N/A	N/A	N/A	N/A
Cs_FN_Ch05_110	7,533	CM011609.1	76,119,680	76,127,213	633	40S ribosomal protein Sa-1	ONL94284.1	21	7e-19	91.30	*
					519	Putative viral resistance protein	BAD53862.1	35	0.96	35.94	*
Cs_FN_Ch06_111	6,924	CM011610.1	21,831,999	21,838,923	1791	Cannabidiolic acid synthase	AJB28530.1	87	0.0	98.66	***
Cs_FN_Ch06_112	57,056	CM011610.1	21,980,060	22,037,116	612	Retrovirus-related pol polyprotein from transposon TNT 1-94	RVW52634.1	83	2e-70	70.59	*
					318	Hypothetical protein Saspl_048356	TEY18652.1	41	3.6	46.81	*
					522	Splicing factor suppressor of ABI3-5	PNX97210.1	41	8.7	33.33	*
					348	Pol polyprotein	RDX76937.1	61	2e-18	49.30	*
					3303	Uncharacterized protein LOC104901504	XP_010687394.1	58	2e-116	31.59	*
Cs_FN_Ch07_113	2,870	CM011611.1	75,606,412	75,609,282	156	Poly(ADP-ribose) polymerase	PON43801.1	98	4e-20	86.00	*
Cs_FN_Ch10_114	1,184	CM011614.1	33,220,348	33,221,532	324	DJ-1 superfamily protein	PON82055.1	100	4e-63	88.89	*

Supplementary Table S3. Candidate genes identified from CBDA and THCVA pools using the FN reference sequence (*cont.*).

Interval (locus)	Length (bp)	Chr.	Start coordinate	Stop coordinate	Gene length (bp)	Predicted protein[1]	GenBank accession	Cover (%)	*E*-value	Identity (%)	Relevance[2]
Cs_FN_SS00_115	36,516	Super_Scaffold0	8,512,398	8,548,914	2049	Retrovirus-related pol polyprotein from transposon 17.6	RVW67055.1	66	0.0	62.45	*
					1293	Transposon Ty3-I Gag-Pol polyprotein	RVW79670.1	95	2e-146	47.73	*
					432	Hypothetical protein PAHAL_3G132000	PVH61823.1	27	3.8	37.50	*
					2934	Hypothetical protein AMTR_s05719p00002740	ERM97918.1	31	5e-153	89.21	*
					1449	Protein FAR1-RELATED SEQUENCE 4	XP_006847242.1	95	0.0	99.56	**
					747	Zinc finger, CCHC-type	POO00252.1	50	8e-18	32.26	**
					159	Cytochrome P450 87A3	XP_021863246.1	38	1.6	50.00	*
Cs_FN_SS00_116	22,630	Super_Scaffold0	8,575,175	8,597,805	456	Retrovirus-related Gag/Pol protein	ADJ18449.1	36	2e-07	47.27	*
					1110	Retrovirus-related pol polyprotein from transposon TNT 1-94	RVW52633.1	100	1e-156	54.06	*
					399	Putative RNA-directed DNA polymerase	PRQ55763.1	49	3e-14	49.23	*
					840	Putative ribonuclease H protein	RVX04314.1	85	3e-28	32.64	*
					789	LRR receptor-like kinase family protein	KEH19857.1	14	0.030	57.89	*
					192	Uncharacterized protein LOC109158404	XP_019161859.1	53	9.4	35.29	*
					3042	Transposon Ty3-G Gag-Pol polyprotein	RVW68203.1	77	0.0	51.30	*
					1218	Peptide methionine sulfoxide reductase	PON80230.1	30	1e-17	35.77	*
					720	MND1-interacting protein 1	XP_015612542.1	34	4.9	34.15	*
Cs_FN_SS00_117	1,287	Super_Scaffold0	8,641,444	8,642,731	1050	Probable serine/threonine-protein kinase fhkB isoform X2	XP_021985327.1	18	9.5	26.80	*
Cs_FN_SS00_118	2,849	Super_Scaffold0	8,692,374	8,695,223	810	Hypothetical protein AMTR_s01250p00009040	ERM98296.1	27	3e-10	53.09	*
Cs_FN_SS00_119	11,833	Super_Scaffold0	9,039,053	9,050,886	3339	Transposon Ty3-I Gag-Pol polyprotein	RVW67492.1	89	0.0	50.45	*
Cs_FN_SS00_120	4,461	Super_Scaffold0	9,052,611	9,057,072	1050	Transposon Tf2-2 polyprotein	RVW68715.1	91	3e-123	45.04	*
					291	Wall-associated receptor kinase-like 4	XP_023645700.1	58	2.6	29.03	*
Cs_FN_SS00_121	2,062	Super_Scaffold0	39,955,730	39,957,792	150	Uncharacterized protein LOC104714925	XP_010430691.1	97	6.1	97%	*
Cs_FN_SS00_122	2,817	Super_Scaffold0	147,482,669	147,485,486	243	Sugar transport protein 5-like isoform X1	XP_015692118.1	97	8.4	29.63	*

[1]Protein prediction based on alignment with non-redundant sequences using the National Center for Biotechnology Information (NCBI) Basic Local Alignment Search Tool (BLAST)p algorithm. Protein selection based on highest scoring characterised protein, then identity, then coverage, then *E* value. [2]Relevance to chemotype: *: unclear; **: possible; ***: likely

Supplementary Table S4. Candidate genes identified from CBDA and THCVA DNA pools using the PK reference sequence.

Locus (interval)	Length (bp)	Chr.	Start coordinate	Stop coordinate	Gene length (bp)	Predicted protein	GenBank accession	Cover (%)	*E*-value	Identity (%)	Relevance
Cs_PK_Ch02_101	27,447	CM010792.1	34,376,781	34,404,228	711	Hypothetical protein PHAVU_009G169400g	XP_007137962.1	30	9.8	32.00	*
					465	Reverse transcriptase	OMO58663.1	46	9.7	35.14	*
					411	Uncharacterized protein LOC8280609	XP_015573636.1	29	6.4	35.00	*
					558	Transposon Tf2-8 polyprotein	RVW92564.1	44	4e-25	51.76	*
					1185	Zf-CCHC domain-containing protein/UBN2 domain-containing protein	GAV67439.1	99	2e-76	39.34	*
					273	WAT1-related protein At5g64700-like	XP_027074243.1	41	1.2	41.86	*
Cs_PK_Ch02_102	43,208	CM010792.1	60,414,035	60,457,243	1686	Transposase	BAV56702.1	34	1e-38	41.33	*
					369	Hypothetical protein PAHAL_3G132000	PVH61823.1	31	5.6	36.84	*
					1074	Ribonuclease H-like domain containing protein	PON53148.1	49	3e-97	79.10	*
					708	Ribonuclease H-like domain containing protein	PON53148.1	84	1e-72	55.14	*
					798	Ulp1 protease family, C-terminal catalytic domain containing protein	PON68164.1	35	0.027	29.17	*
					150	40S ribosomal protein Sa-2	XP_002303327.2	55	7e-09	92.59	*
					3432	Beta-porphyranase	PSR88450.1	43	2e-63	31.14	*
					459	Ribonuclease H-like domain containing protein	PON92320.1	38	3e-15	60.34	*
					1512	Peptide methionine sulfoxide reductase	PON80230.1	38	3e-27	34.17	*
Cs_PK_Ch03_103	1,183	CM010793.1	73,374,622	73,375,805	414	Pleiotropic drug resistance protein 2-like isoform X3 [Prunus avium]	XP_021818809.1	89	3e-58	76.42	*
Cs_PK_Ch06_104	3772	CM010796.1	59,095,674	59,099,446	N/A	No gene predicted for interval	N/A	N/A	N/A	N/A	N/A
Cs_PK_Ch07_105	1,510	CM010797.1	1,666,404	1,667,914	N/A	No gene predicted for interval	N/A	N/A	N/A	N/A	N/A
Cs_PK_Ch09_106	2,835	CM010799.1	49,163,072	49,165,907	915	Beta-ketoacyl-ACP reductase	XP_003538564.1	100	4e-136	67.74	***
Cs_PK_Ch09_107	12,046	CM010799.1	49,166,568	49,178,614	201	FkbH domain-containing protein	PON89298.1	80	9e-19	77.36	***
Cs_PK_Ch09_108	12,365	CM010799.1	49,179,279	49,191,644	549	TRAM/LAG1/CLN8 domain-containing protein (TLC domain)	PON82787.1	99	9e-101	81.77	***
					1575	DNA polymerase alpha, subunit B	PON82786.1	94	0.0	88.00	*
Cs_PK_Ch09_109	1,340	CM010799.1	49,190,198	49,191,538	369	DNA polymerase alpha subunit B	XP_012085629.1	100	5e-35	56.91	*

[1]Protein prediction based on alignment with non-redundant sequences using the National Center for Biotechnology Information (NCBI) Basic Local Alignment Search Tool (BLAST)p tool. Protein selection based on highest scoring characterised protein, then identity, then coverage, then *E* value. [2]Relevance to chemotype: *: unclear; **: possible; ***: likely

Supplementary Table S5. *BKR* short variants putatively linked to alkyl cannabinoid composition.

Locus ID	Ref allele	Alt allele	Region	Amino acid change	Position on Chr. 9	CBDA pool AFe	CBDA pool DP	THCVA pool AFe	THCVA pool DP	Delta-AFe
Cs_BKR_02	A	G	Intron	N/A	49162146	0.1	62	1.0	45	0.9
Cs_BKR_02	A	C	Intron	N/A	49162180	0.0	62	1.0	41	1.0
Cs_BKR_03	A	G	Intron	N/A	49162335	0.0	66	1.0	40	1.0
Cs_BKR_04	T	G	Intron	N/A	49162379	0.1	71	1.0	41	0.9
Cs_BKR_05	A	G	Intron	N/A	49162529	0.0	53	1.0	59	1.0
Cs_BKR_06	G	A	Exon	Ala to Val	49162975	0.1	62	1.0	65	0.9
Cs_BKR_07	T	C	Exon	Ser to Asn	49163005	0.1	61	1.0	64	0.9
Cs_BKR_08	A	G	Intron	N/A	49163072	0.0	52	0.9	62	0.9
Cs_BKR_09	A	G	Intron	N/A	49163190	0.0	57	1.0	47	1.0
Cs_BKR_10	A	G	Intron	N/A	49163323	0.0	76	1.0	53	1.0
Cs_BKR_11	AT	A	Intron	N/A	49163486	0.0	77	1.0	42	1.0
Cs_BKR_12	T	C	Intron	N/A	49163595	0.1	66	1.0	56	0.9
Cs_BKR_13	G	T	Intron	N/A	49163650	0.0	71	1.0	64	1.0
Cs_BKR_14	T	C	Intron	N/A	49163849	0.0	68	0.9	47	0.9
Cs_BKR_15	T	C	Intron	N/A	49163850	0.0	67	0.9	47	0.9
Cs_BKR_16	A	G	Intron	N/A	49163871	0.0	66	1.0	41	1.0
Cs_BKR_17	A	AC	Intron	N/A	49165872	0.0	79	1.0	91	1.0
Cs_BKR_18	C	T	Exon	N/A	49165925	0.1	77	1.0	97	0.9
Cs_BKR_19	G	A	Exon	N/A	49165943	0.1	76	1.0	105	0.9
Cs_BKR_20	ATTG	A	5' UTR	N/A	49166087	0.0	72	1.0	155	1.0
Cs_BKR_21	C	A	5' UTR	N/A	49166135	0.0	63	0.9	177	0.9
Cs_BKR_22	A	C	5' UTR	N/A	49166141	0.0	63	0.9	181	0.9

AFe: is the relative number of reads supportive of either the alternative allele or the reference allele. AFe of 0.0 indicates 100 % of reads support the reference allele. AFe 0f 1.0 indicates 100 % of reads support the alternative allele. AFe: allele frequency estimates; delta-AFe: absolute difference between AFe of CBDA and THCVA pools; DP: depth of coverage; Chr 9: GenBank Acc. CM010799.1.

Supplementary Table S6. Alignment of putative alkyl cannabinoid candidate genes with the PK draft transcriptome.

Putative gene homolog	Query length	Target mRNA sequence	Query Start	Query Finish	Query Cover (%)	Query Identity (%)	*E*-value
BKR	915	PK02233.1	422	908	53	100	8.9e-281
		PK02233.1	168	351	20	100	5.8e-100
		PK02233.1	1	169	18	99	7.8e-087
		PK03757.1	250	297	5	100	4.7e-019
		PK03757.1	296	343	5	100	4.7e-019
		PK03827.1	275	347	7	90	2.9e-017
fkbH	201	PK27448.1	10	181	85	100	1.7e-093
		PK21994.1	23	181	74	92	6.3e-058
TLC	549	PK20880.1	1	549	100	100	0.000000

Alignment of mRNA sequences with the canStat3 Purple Kush draft transcriptome was performed using the TimeLogic® Tera-BlastN™ algorithm (Active Motif Inc.). *E*-value cut-off limit 1e-10. The canSat3_transcriptome-representative.fa file was downloaded from the *Cannabis* Genome Browser (date accessed 12/04/2019): http://genome.ccbr.utoronto.ca/downloads.html
